# Short-Term Effect of Pollen and Spore Exposure on Allergy Morbidity in the Brussels-Capital Region

**DOI:** 10.1007/s10393-016-1124-x

**Published:** 2016-05-12

**Authors:** Ariane Guilbert, Koen Simons, Lucie Hoebeke, Ann Packeu, Marijke Hendrickx, Koen De Cremer, Ronald Buyl, Danny Coomans, An Van Nieuwenhuyse

**Affiliations:** 1 Health and Environment Unit, Scientific Institute of Public Health, Rue Juliette Wytsmanstraat 14, 1050 Brussels, Belgium; 2 Mycology and Aerobiology Unit, Scientific Institute of Public Health, Rue Juliette Wytsmanstraat 14, 1050 Brussels, Belgium; 3 Department of Biostatistics and Medical Informatics - Public Health, Vrije Universiteit Brussel, Laarbeeklaan 103, 1090 Brussels, Belgium

**Keywords:** allergic rhinitis, ecology, fungal spore, medication, pollen, public health

## Abstract

Belgium is among the European countries that are the most affected by allergic rhinitis. Pollen grains and fungal spores represent important triggers of symptoms. However, few studies have investigated their real link with disease morbidity over several years. Based on aeroallergen counts and health insurance datasets, the relationship between daily changes in pollen, fungal spore concentrations and daily changes in reimbursable systemic antihistamine sales has been investigated between 2005 and 2011 in the Brussels-Capital Region. A Generalized Linear Model was used and adjusted for air pollution, meteorological conditions, flu, seasonal component and day of the week. We observed an augmentation in drug sales despite no significant increase in allergen levels in the long term. The relative risk of buying allergy medications associated with an interquartile augmentation in pollen distributions increased significantly for Poaceae, *Betula*, *Carpinus*, *Fraxinus* and *Quercus*. Poaceae affected the widest age group and led to the highest increase of risk which reached 1.13 (95% CI [1.11–1.14]) among the 19- to 39-year-old men. *Betula* showed the second most consistent relationship across age groups. Clear identification of the provoking agents may improve disease management by customizing prevention programmes. This work also opens several research perspectives related to impact of climate modification or subpopulation sensitivity.

## Introduction

Allergic rhinitis is a chronic inflammatory disease of the upper airways caused by an IgE-mediated reaction. Through many nose and throat symptoms such as sneezing, itchiness, nasal congestion or discharge, it significantly impairs patients’ quality of life (Bousquet et al. 2008). According to the World Allergy Organization, it touched between 10 and 30% of the world population in 2011 (Pawankar et al. 2011). Belgium seems particularly affected. In a cross-sectional study carried out by Bauchau and Durham in six European countries in 2001, this country showed the highest prevalence: 28,5% (Bauchau and Durham 2004).

Symptoms can be triggered by indoor allergens including pets’ dander, house dust mite and/or outdoor allergens such as pollen grains and fungal spores. These last two categories are responsible for the seasonal symptom expression commonly observed (Pedersen and Weeke 1984; Bousquet et al. 2008). Air pollution plays also a key role, tending to worsen allergic rhinitis symptoms (Hajat et al. 2001). More generally, global environmental change such as increase in temperature and CO_2_ concentration, new territory planning strategies, introduction of new species, are likely to affect patients by modifying distribution, concentration, season, allergenicity of pollen grains but also potentially mould spores (Beggs 2004; Reid and Gamble 2009).

Exploring provoking agents more closely may help to improve disease management. However, few studies have investigated this issue and the methodology was rather diverse. While some analysed the association between pollen levels and medication sales (Laaidi 2000; Christophe et al. 2003; Ravault et al. 2005; Sánchez-Mesa et al. 2005; Zeghnoun et al. 2005; Fuhrman et al. 2007; Johnston et al. 2009; Sheffield et al. 2011; Van Vliet and Tobi 2012; Motreff et al. 2013; Caillaud et al. 2015) or medical visits (Pedersen and Weeke 1984; Breton et al. 2006; Zhang et al. 2012), others looked at emergency, department visits (Cakmak et al. 2002), focusing on the most severe cases. Whereas some explored the relationship with time series designs (Cakmak et al. 2002; Ravault et al. 2005; Zeghnoun et al. 2005; Breton et al. 2006; Fuhrman et al. 2007; Johnston et al. 2009; Sheffield et al. 2011; Van Vliet and Tobi 2012; Zhang et al. 2012; Motreff et al. 2013; Caillaud et al. 2015), others used descriptive or correlation methods (Pedersen and Weeke 1984; Laaidi 2000; Christophe et al. 2003; Sánchez-Mesa et al. 2005), failing to adjust for confounders. In addition, local context including vegetation, topography, physician prescription behaviour, etc. influences results and limits extrapolation to other regions. Based on time series analysis and restricting to the European context, a few studies highlighted the relationship between allergy medication sales and *Alnus*, *Betula*, *Carpinus*, *Corylus*, Cupressaceae, *Fraxinus*, Poaceae (also referred to as Gramineae), *Olea*, Plantaginaceae, *Platanus*, *Quercus*, *Salix*, Urticaceae pollen levels (Ravault et al. 2005; Zeghnoun et al. 2005; Fuhrman et al. 2007; Van Vliet and Tobi 2012; Motreff et al. 2013; Caillaud et al. 2015). However, more than half of the studies concentrated on the same geographical area and data collection methods did not cover the whole studied population.

This study aimed to assess, for the first time in the Brussels-Capital Region (BCR) and in Belgium, the short-term relationship between pollen levels and allergy medication sales based on extensive administrative datasets. It also investigated the role of outdoor fungal spores which received little attention despite their high allergenic properties (Tariq et al. 1996; Andersson et al. 2003).

## Methods

### Settings

The research has been carried out in the BCR. This urban area is composed of 19 municipalities representing approximately 1,175,000 inhabitants spread over 161 km^2^. Allergen concentrations, air pollution, weather, health behaviours and medical practices were all deemed sufficiently homogeneous over this sector. The studied time period covers seven pollen seasons, from 2005 to 2011 included.

### Exposure Measures

The analysis investigated the impact of nine pollen types (*Alnus*, *Betula*, *Carpinus*, *Corylus*, *Fraxinus*, Poaceae, *Quercus*, *Taxus* combined with Cupressaceae) and two fungal spore types (*Alternaria* and *Cladosporium*). This selection was based on the allergenicity and distribution of these substances in the BCR. Their daily concentrations were provided by the Mycology and Aerobiology service (www.airallergy.be) of the Belgian Scientific Institute of Public Health (WIV-ISP). The counts of pollen and fungal spores in the air were obtained using a Burkard© volumetric spore sampler located on the top of the WIV-ISP building, in the centre of the study area. Measurements were carried out 7 days a week, from January to November included. For the month of December, zeros were imputed for all types. This decision was based on experience: regular pollen counts have shown that by the end of September, allergenic pollen concentrations become close to zero. A similar trend is observed for fungal spores, whose concentrations drastically decrease and become too low to trigger symptoms in November.

### Outcome Measures

The health impact of pollen grains and fungal spores was assessed based on their association with reimbursable “Antihistamines for systemic use” sales (R06A category according to the World Health Organization Anatomical Therapeutic Chemical classification system [ATC]; detailed list available as supplemental material), referred to as “allergy medications” hereinafter. Figures were obtained from the Pharmanet database, managed by the InterMutualistic Agency. This administrative system records, on a daily basis, data on any purchase of refundable drugs made by a patient affiliated to the social security, in one of the public Belgian pharmacies. It contains for each drug sale: information on product code, quantity, day of purchase and customer through an encoded social security number. This identification number enabled merging of the Pharmanet records with the Population database. The latter, also managed by the InterMutualistic Agency, includes data on customer sociodemographic characteristics (age, gender, place of residence). Because health insurance is mandatory in Belgium, these databases are virtually complete. For this study, sales data were restricted to people residing in the BCR at the time of purchase.

### Confounding Factors

Among air pollutants, PM_10_, NO_2_, SO_2_ and O_3_ were considered as potential confounders. The Belgian Interregional Communication Cell for the Environment provided daily mean concentrations for the BCR. These data are derived from a national monitoring network (11 stations in the BCR), augmented by a land-use regression model: RIO-CORINE (Janssen et al. 2008). The influence of meteorological factors including daily minimal temperature and average relative humidity, was taken into account. Data were measured and provided by the Royal Meteorological Institute of Belgium located within the study area. Day of the week, holiday and flu season (binary variable defined by the WIV-ISP in partnership with a representative network of sentinel general practitioners) were also introduced in the statistical model as cofactors.

### Statistical Analysis

Sales records were aggregated into ecological time series. A Generalized Linear Model based on a Poisson distribution with log-link and corrected for overdispersion was used (McCullagh and Nelder 1989). Single pollen and spore models were fit, using an unrestricted distributed lag of zero to 10 days (Almon 1965). For each pollutant, a daily average estimate over the study area was calculated (local concentrations weighted by the total population per cell). Because of the high intercorrelation between these compounds, the model was only corrected for same day PM_10_. Sensitivity analyses showed similar results when correcting for same day NO_2_, etc. Based on prior work and sensitivity analysis, cubic splines with seven degrees of freedom per year were used to adjust for season and trend. Daily minimum temperature and daily average relative humidity were modelled with natural cubic splines of, respectively six and three degrees of freedom. Flu season was introduced as a binary variable per season. Day of the week was also considered. Pharmacies are all open on weekdays; however, in the weekend and on holidays (official days off), access is diminished which results in low or zero sale volumes. Thus, only weekdays provide valuable information and other days were removed from the outcome series. As pollen sensitivity and health behaviour may vary according to age, analyses were performed for different age groups (0–5, 6–12, 13–18, 19–39, 40–64, 65–84 and 85 years or more). Impact of gender was also considered. All analysis were carried out using R 3.1.0^©^ (R Core Team 2015). This study has been approved by the Belgian Commission for the Protection of Privacy.

## Results

### Pollen and Fungal Spore Exposure

**Figure 1:**
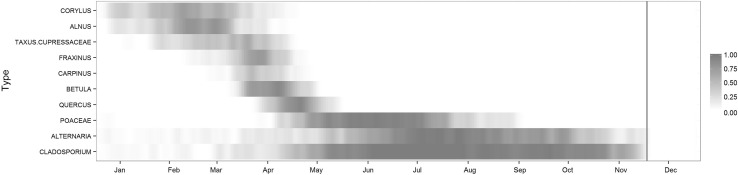
Descriptive statistics on pollen, spore levels, BCR, 2005–2011. Colour intensity represents the probability of observing pollen or spore counts larger than 1% of the type’s maximum concentration. The vertical line highlights the month from which pollen and spore counts are considered equal to zero

Exposure period ranged from January to the beginning of September for pollen grains and from April to November for fungal spores. The distribution of allergen concentrations varied greatly between types and from 1 year to another for the same type (especially for *Carpinus* and *Fraxinus*). Maximal concentrations fluctuated between 167 grains/m^3^ for *Corylus* and 43,230 spores/m^3^ for *Cladosporium*. Results are summarized in Fig. 1 and Table 1.

**Table 1:** Descriptive Data on Pollen and Fungal Spore Levels, BCR, 2005–2011

	Annual airborne pollen/spore counts			Contribution* to annual airborne pollen load (%)			Maximum daily airborne pollen/spore count
	Min	Median	Max	Min	Median	Max	
*Corylus*	278	557	1743	1.43	2.19	6.05	167
*Alnus*	563	1804	2434	3.52	6.35	9.26	342
*Taxus* Cupressaceae	3465	5525	10,757	19.37	25.73	32.25	2595
*Fraxinus*	242	1419	3360	1.04	7.40	10.54	728
*Carpinus*	29	285	1001	0.18	1.00	4.98	387
*Betula*	3740	8240	12,141	25.34	35.30	44.92	2464
*Quercus*	760	2371	5335	3.90	10.95	18.53	560
Poaceae	1926	2589	3649	7.55	9.89	18.42	246
*Alternaria*	10,435	21,520	29,210	/	/	/	1985
*Cladosporium*	667,775	736,860	1,122,660	/	/	/	43,230

*Contribution calculated as ratio of type count over the sum of all pollen types listed in the table

### Medication Sales

1,370,535 reimbursable allergy medication purchases by 347,034 distinct individuals were recorded between 2005 and 2011. Table 2 provides descriptive information on allergy medication sales. The majority of patients (62.8%) bought only one box of reimbursed allergy medications per year, 78.9% bought two or less, 97.7% bought less than ten boxes.

**Table 2:** Distribution of Daily Number of Sales of Eligible Allergy Medication (Number of Boxes Per Day), BCR, 2005–2011

Age	Gender	Min	Max	Mean	Median	1st quartile	3rd quartile
0-5 years	Male	1	42	16.4	16	12	20
	Female	1	39	12.8	12	9	16
6-12 years	Male	2	68	21.2	20	15	26
	Female	3	42	15.9	15	12	19
13-18 years	Male	1	48	14.6	13	10	18
	Female	1	48	14.3	13	10	17
19-39 years	Male	20	218	58.9	50	41	68
	Female	41	308	96.0	82	70	113
40-64 years	Male	29	203	86.3	79	67	100
	Female	68	396	165.0	148	131	189
65-84 years	Male	23	105	54.5	54	46	62
	Female	25	191	105.0	102	89	118
85 or more years	Male	0	30	9.1	9	6	11
	Female	12	103	32.2	31	26	36

Figures calculated excluding Saturday, Sunday and holidays

Table 3 shows the percentage of residents buying at least one eligible product in a given year. For most age groups, percentages increased (non-consistently) between 2005 and 2011. The 13–18 years and 40–64 years groups showed the highest increase (up to 21% for men 40–64 years old). In general, purchases were more frequent among males at early age (0-12 years) while females were more likely to buy allergy medications from age 13. Male/female prevalence ratio varied between 1.18 for 6–12 years group and 0.58 for 40–64 years group.

**Table 3:** Percentage of Inhabitants Buying at Least One Eligible Allergy Drug, BCR, 2005–2011

Age	Gender	2005	2006	2007	2008	2009	2010	2011
0-5 years	Male	8.95	8.47	8.13	7.86	7.16	6.87	7.22
	Female	7.83	7.18	6.81	6.61	5.96	6.00	6.34
6-12 years	Male	8.13	8.30	8.20	8.14	8.48	8.05	8.30
	Female	6.97	6.94	6.92	7.03	7.10	6.90	6.80
13-18 years	Male	6.25	6.63	7.07	7.33	7.57	7.00	7.21
	Female	6.95	7.07	7.26	8.00	7.69	7.67	8.05
19-39 years	Male	5.72	5.87	5.93	6.25	6.25	6.04	6.12
	Female	9.08	9.31	9.52	9.87	9.76	9.59	9.66
40-64 years	Male	6.94	7.22	7.24	8.23	8.36	8.40	8.38
	Female	12.55	13.02	13.09	14.02	14.11	13.74	14.22
65-84 years	Male	9.94	10.34	10.34	10.99	10.84	10.36	10.23
	Female	13.06	13.18	13.30	13.73	13.73	13.46	13.50
85 or more years	Male	10.46	10.20	10.16	10.51	10.62	10.84	11.02
	Female	12.22	12.41	12.19	12.58	12.63	12.28	12.03

### Relationship Between Pollen, Fungal Spore Exposure and Medication Sales

**Figure 2:**
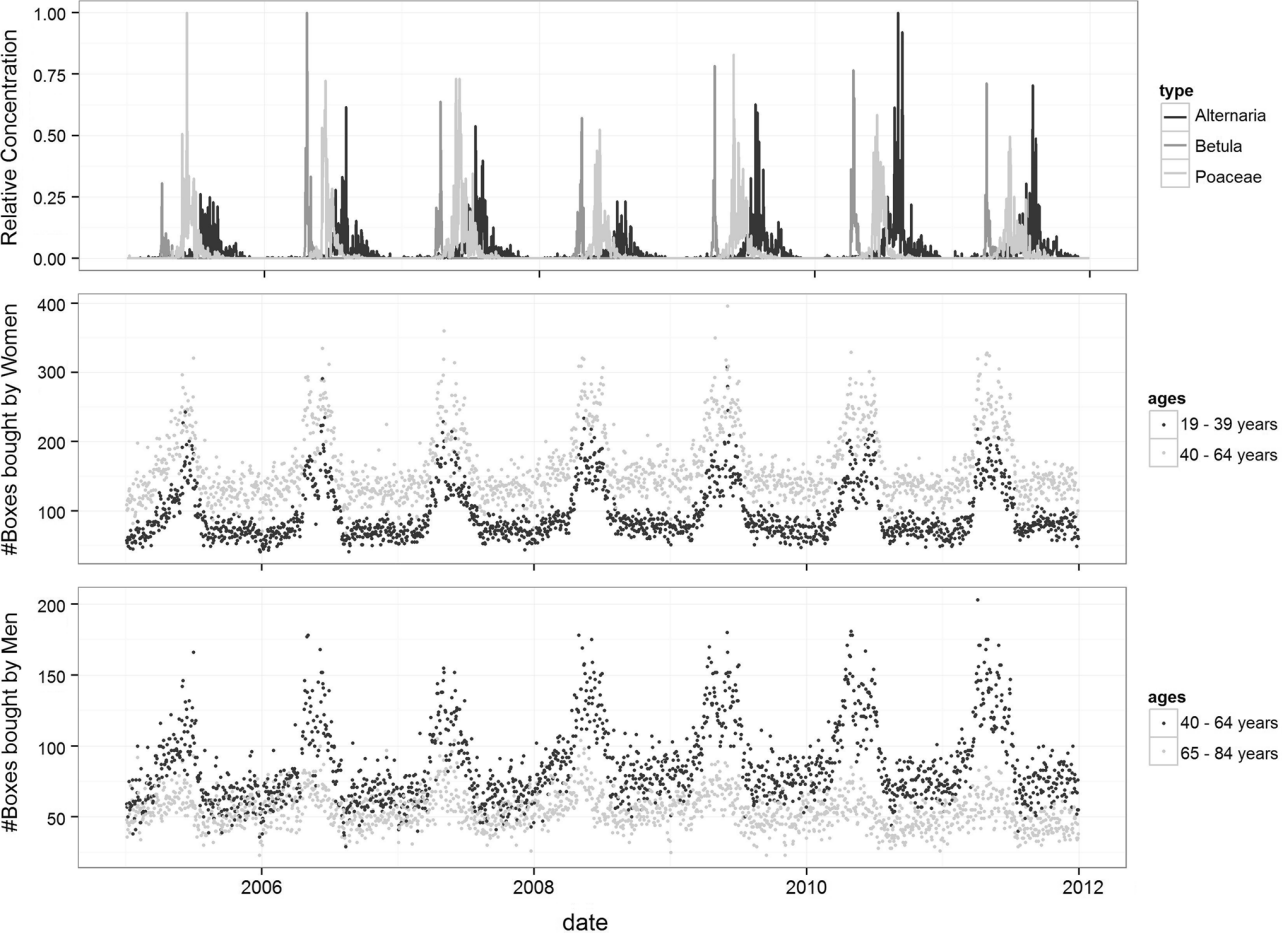
Selected time series, BCR, 2005–2011. Top: daily pollen concentrations of *Alternaria*, *Betula* and Poaceae, scaled (divided by the maximum concentration observed for these three types). Middle: Daily number of boxes of eligible medications purchased by women, ages 19–39 and 40–64 (excluding Saturdays, Sundays and holidays). Bottom: Daily number of boxes of eligible medications purchased by men, ages 40–64 and 65–84 (excluding Saturdays, Sundays and holidays)

Figure 2 shows the temporal evolution of several pollen, fungal spore concentrations and allergy medication sales. For readability, only three aeroallergens (*Alternaria*, *Betula* and Poaceae) and three age groups (19–39, 40–64 and 65–84 years) are displayed based on their relevance. Overall, medication sales exhibited a high seasonality with a noteworthy peak between April and June. These variations tended to be less and less pronounced with increasing age. Men and women followed a similar pattern.

The short-term association between pollen, spore levels and allergy medication sales has been investigated for a lag zero to 10 days, adjusting for the various confounders. Results are expressed as the relative risk of buying allergy medications associated with an interquartile augmentation (InterQuartile Range [IQR]) in pollen or spore distributions with zero to 10 days lag. They are summarized in Table 4. Considering the overall population, risks were significantly increased for Poaceae, *Betula*, *Carpinus*, *Fraxinus* and *Quercus*. No significant association was observed for *Corylus*, *Alnus* and *Taxus* Cupressaceae. Differences across age were manifest. From 0 to 5 years, the risk only increased for Poaceae. From 6 to 12 years, the risk was positive for Poaceae but also for *Betula* and *Carpinus*. From 13 to 18 years, Poaceae*, Betula* and *Quercus* were associated with an increasing risk. From 19 to 64 years, the risk increased for Poaceae*, Betula, Carpinus*, *Fraxinus* and *Quercus*. The latter specifically affected individuals aged from 65 to 84 years. No positive association was demonstrated for the oldest group (85 years or more). When restricting to pollen, the significant relative risks per IQR ranged from 1.02 (95% CI [1.01–1.03]) due to *Fraxinus* to 1.13 (95% CI [1.11–1.14]) due to Poaceae. Gender impacted results with no clear pattern according to allergen types. Associations seemed a bit stronger for men than women among the 19–64 years group. Regarding *Alternaria* and *Cladosporium* spores, a significantly negative risk was observed for most of the age and gender groups.

**Table 4:** Relative Risk (95% confidence interval) of Buying Reimbursable Allergy Medications Associated with an Interquartile Range Increase in Pollen or Spore Distribution

Pollen and spore types		*Corylus*	*Alnus*	*Taxus* Cupressaceae	*Fraxinus*	*Carpinus*	*Betula*	*Quercus*	Poaceae	*Alternaria*	*Cladosporium*
Interquartile range (count per m^3^)^†^		9	18.25	16	24.5	7	84.75	68	20	90	2965
0–5 years	Male	0.97 (0.94–1.00)	1.00 (0.98–1.03)	1.00 (0.99–1.00)	0.99 (0.97–1.01)	1.02 (0.99–1.05)	1.01 (1.00–1.03)	1.00 (0.97–1.04)	1.05 (1.02–1.08)*	0.93 (0.90–0.96)*	0.97 (0.94–1.00)
	Female	0.97 (0.93–1.01)	1.00 (0.97–1.02)	1.00 (0.99–1.00)	1.00 (0.98–1.02)	1.01 (0.98–1.04)	1.00 (0.99–1.02)	0.99 (0.95–1.03)	1.07 (1.04–1.10)*	0.92 (0.89–0.95)*	0.96 (0.93–0.99)*
6–12 years	Male	0.99 (0.96–1.02)	1.00 (0.98–1.02)	1.00 (0.99–1.01)	1.02 (1.00–1.03)	1.03 (1.00–1.06)	1.02 (1.01–1.04)*	0.98 (0.95–1.01)	1.10 (1.07–1.13)*	0.89 (0.87–0.92)*	0.92 (0.90–0.95)*
	Female	0.98 (0.95–1.02)	0.98 (0.96–1.01)	1.00 (0.99–1.00)	1.01 (0.99–1.03)	1.03 (1.01–1.06)*	1.02 (1.00–1.03)	0.99 (0.96–1.03)	1.09 (1.07–1.12)*	0.89 (0.87–0.92)*	0.93 (0.91–0.96)*
13–18 years	Male	1.00 (0.96–1.03)	1.00 (0.98–1.03)	1.00 (0.99–1.01)	1.01 (0.98–1.03)	1.03 (1.00–1.06)	1.03 (1.01–1.04)*	1.02 (0.99–1.06)	1.07 (1.04–1.10)*	0.90 (0.87–0.93)*	0.92 (0.89–0.95)*
	Female	1.00 (0.96–1.04)	1.01 (0.98–1.04)	1.00 (0.99–1.01)	1.02 (0.99–1.04)	1.03 (1.00–1.06)	1.02 (1.01–1.04)*	1.05 (1.01–1.09)*	0.99 (0.97–1.02)	0.92 (0.89–0.95)*	0.95 (0.92–0.98)*
19–39 years	Male	1.00 (0.98–1.03)	1.00 (0.98–1.01)	1.01 (1.00–1.01)	1.05 (1.04–1.06)*	1.08 (1.06–1.09)*	1.05 (1.04–1.06)*	0.96 (0.94–0.98)*	1.13 (1.11–1.14)*	0.95 (0.93–0.97)*	0.94 (0.93–0.96)*
	Female	0.99 (0.98–1.01)	0.99 (0.98–1.01)	1.00 (1.00–1.01)	1.03 (1.02–1.04)*	1.05 (1.03–1.06)*	1.03 (1.02–1.04)*	0.99 (0.97–1.01)	1.10 (1.08–1.11)*	0.94 (0.93–0.96)*	0.95 (0.93–0.96)*
40–64 years	Male	0.99 (0.98–1.01)	1.00 (0.99–1.01)	1.00 (1.00–1.01)	1.03 (1.02–1.04)*	1.06 (1.04–1.07)*	1.04 (1.03–1.04)*	1.00 (0.99–1.02)	1.06 (1.04–1.07)*	0.94 (0.93–0.96)*	0.96 (0.95–0.98)*
	Female	0.99 (0.98–1.01)	1.00 (0.99–1.01)	1.00 (1.00–1.00)	1.02 (1.01–1.03)*	1.03 (1.02–1.04)*	1.03 (1.02–1.03)*	1.03 (1.01–1.04)*	1.04 (1.03–1.06)*	0.95 (0.94–0.96)*	0.97 (0.95–0.98)*
65–84 years	Male	0.99 (0.97–1.01)	1.00 (0.98–1.01)	1.00 (1.00–1.00)	1.00 (0.99–1.01)	1.00 (0.98–1.01)	1.00 (1.00–1.01)	1.03 (1.01–1.05)*	1.01 (0.99–1.03)	1.00 (0.98–1.01)	0.97 (0.95–0.99)*
	Female	0.99 (0.97–1.00)	1.00 (0.99–1.01)	1.00 (1.00–1.00)	1.00 (0.99–1.01)	1.00 (0.99–1.02)	1.01 (1.00–1.02)	1.03 (1.01–1.04)*	1.00 (0.99–1.01)	0.98 (0.97–1.00)	0.98 (0.97–0.99)*
85 or more years	Male	0.99 (0.94–1.03)	0.97 (0.94–1.00)	1.00 (0.99–1.00)	0.99 (0.96–1.02)	0.95 (0.92–1.00)	0.99 (0.97–1.01)	1.02 (0.97–1.08)	0.98 (0.94–1.03)	0.97 (0.93–1.01)	0.98 (0.94–1.02)
	Female	0.97 (0.94–1.00)	0.98 (0.96–1.01)	1.00 (0.99–1.00)	1.00 (0.98–1.02)	0.98 (0.95–1.01)	1.00 (0.98–1.01)	1.03 (0.98–1.07)	0.99 (0.96–1.02)	0.97 (0.94–0.99)*	0.96 (0.93–0.99)*

Sum of effects of lags 0 to 10, by age and sex, BCR, 2005–2011

* Significant (P < 0.05)

^†^ Calculated on days with non-zero pollen concentrations

## Discussion

This time series analysis investigated the association between pollen and fungal spore counts and allergy medication sales at the BCR scale. Nine pollen (*Alnus*, *Betula*, *Carpinus*, *Corylus*, *Fraxinus*, Poaceae, *Quercus*, *Taxus* combined with Cupressaceae) and two spore (*Alternaria*, *Cladosporium*) types were tested, taking into account the confounding effect of air pollution, weather and flu.

Data on allergy medication sales were used to assess the 2011 prevalence of treated allergic rhinitis (percentage of residents buying at least one eligible product in a given year). This varied between 6.12% and 14.22% according to age and gender group. These results are consistent with Bauchau and Durham who estimated that 11.9% of the Belgian population used medication for allergic rhinitis in 2001. These figures can be compared with the percentage of individuals self-aware of their disease which reached 20.5% and the percentage of clinically confirmable cases which came to 28.5% (Bauchau and Durham 2004). Another study estimated that 34.1% of the Belgian population is sensitized to at least one common aeroallergen (Bousquet et al. 2007). Despite some approximations related to our methodology (described below), these results suggest a gap between real disease prevalence and patient behaviour.

The prevalence of treated allergic rhinitis increased between 2005 and 2011, especially among teenager and middle age groups who showed a percentage increase up to 21% for men 40–64 years old (from 6.94% in 2005 to 8.38% in 2011). Overall, this rise was much lower than the one observed in England between 2001 and 2005 which reached 45,5% (Ghouri et al. 2008). Reasons for this increase are likely to be multifactorial. They may reflect real evolution of disease prevalence as well as change in medical practice, practitioner or public awareness leading to improved disease management. Because neither pollen nor fungal spore concentrations exhibited a strong increase during the study period, it seems less likely that they contributed to the increase in prevalence. Furthermore, no substantial increase was detected in the decades before the study period (Detandt and Nolard 2000) and the spread of highly allergenic plants such as *Ambrosia* or *Artemisia* remained rather limited in Belgium. In spite of its possible impact on allergic rhinitis prevalence (D’Amato et al. 2001; Hwang et al. 2006), air pollution is also unlikely to be responsible for this disease prevalence increase. Indeed, PM_10_, NO_2_ and O_3_ concentrations did not show substantial variations between 2005 and 2011 and SO_2_ level even decreased.

The prevalence of treated allergic rhinitis varied across demographic factors: while boys appeared more likely to consume allergy medications during childhood, this trend reversed after the puberty, women appearing more affected. This observation may reveal true disparity in sensitization, disease prevalence as already demonstrated (Osman et al. 2007; Govaere et al. 2007) or difference in disease perception and management. Indeed, previous studies have shown that women were more likely to report psychosocial effects due to allergic rhinitis and perceived it as a more threatening disease, leading to a possible greater use of medications (Chen et al. 2008; Pesut et al. 2014).

Positive associations were observed between Poaceae, *Betula*, *Carpinus*, *Fraxinus*, *Quercus* and medication sales. Poaceae affected the widest age group and led to the highest increase of relative risk per IQR (up to 1.13 CI 95% [1.11–1.14] among 19- to 39-year-old men). This family has been recognized as a major cause of pollinosis (D’Amato et al. 2007). In Belgium, it includes around 100 species, with different allergenic potential, which consecutively pollinate resulting in one of the longest pollination season. *Betula* displayed the second most consistent relationship, followed by *Carpinus* and *Fraxinus*. Betula is by far the most allergenic and common tree pollen in Belgium (Detandt and Nolard 2000). It is widely used as ornamental tree and is responsible for around one-third of the total tree pollen load. Its maximal concentration was much higher than the one recorded in France for instance (up to eight times more) (Zeghnoun et al. 2005; Fuhrman et al. 2007). Analogous works showed similar results (Ravault et al. 2005; Zeghnoun et al. 2005; Fuhrman et al. 2007; Sheffield et al. 2011; Van Vliet and Tobi 2012; Caillaud et al. 2015). Associations existed with *Quercus* but were inconsistent. This may be explained by cross-allergenicity phenomenon: *Betula* pollination which occurs just before may have concentrated drug sales. This observation agrees with Fuhrman’s and Caillaud’s results (Fuhrman et al. 2007; Caillaud et al. 2015) but not with other French or American studies (Ravault et al. 2005; Zeghnoun et al. 2005; Sheffield et al. 2011; Motreff et al. 2013). Contrary to what has been observed in France (Ravault et al. 2005; Zeghnoun et al. 2005; Motreff et al. 2013), *Taxus* Cupressaceae levels were not significantly associated with medication sales. Cupressaceae trees are relatively rare in Belgium compared with southern parts of Europe. Sensitivity to this pollen type being conditioned to its abundance, Belgian patients may be subjected to lower concentrations than in France and so be less sensitized (Charpin et al. 1990). This is confirmed by the GA^2^LEN study which highlighted a low sensitization rate to this type in Belgium (Burbach et al. 2009). Regarding *Corylus* and *Alnus*, despite their recognized allergenic potential (Weryszko-Chmielewska et al. 2001; D’Amato et al. 2007), no significant association with medication sales was detected here. The relatively low levels of exposure are unlikely to explain such result as daily concentrations less than 50 grains/m^3^ are sufficient to trigger first symptoms (Weryszko-Chmielewska et al. 2001; Rapiejko et al. 2007). Moreover, Belgian patients do not present a lower sensitivity to these allergens compared with the European average or France where significant associations with medication sales were observed for concentrations of the same order of magnitude (Ravault et al. 2005; Zeghnoun et al. 2005; Fuhrman et al. 2007; Burbach et al. 2009). These pollen types being mainly released during the winter/early spring, one could consider a misdiagnosis between allergies and cold and so the consumption of medications different from antihistamines.

Regarding *Alternaria* or *Cladosporium* spores, a consistent significant negative association with medication sales was observed. In the framework of the European Community Respiratory Health Survey (ECRHS), Bousquet showed low sensitization to these allergens in the Belgian population (less than 2.5%) (Bousquet et al. 2007). Moreover, this result is likely to be related to Poaceae which pollinates just before. The few patients sensitized to these allergens may be already under treatment at the time fungal spores peak. In a similar study carried out in Australia, no significant association was observed between medication sales and total fungal spores (Johnston et al. 2009).

It should be noted that patient susceptibility is likely to vary throughout the year according to pollen, spore concentrations, air pollution levels but also due to physiological reactions. Two panel studies investigated the correlation between Poaceae pollen concentrations and symptoms developed by a sample of sensitized patients (Nolard and Duchaine 1978; Weger et al. 2011). In both cases, patients experienced more severe symptoms in the early season compared to the late season at similar pollen concentrations. These observations were not significantly affected by medication use or co-sensitization to another pollen. This reaction might be explained by a mechanism of priming according to which repeated exposure to pollen may induce tolerance.

When considering age, different profiles appeared. The group 19–64 years was the most affected, responding to the widest group of pollen and showing the highest relative risks. Despite a rather high percentage of individuals buying at least one allergy drug, scarce significant relationships were demonstrated for ages above 64 years. When looking at specific time series for these age groups, it is clear that seasonality is much less pronounced than in the younger populations. Elderly individuals are likely to follow a sales pattern different from the general population due to variations in healthcare use. Indeed, diagnosis is complicated by concurrent diseases provoking similar symptoms and treatment strategy may differ from the general population to limit drug interactions (Busse and Kilaru 2009). In addition, older persons might be less exposed to aeroallergens due to mobility limitations. This absence of association is not always observed in similar works (Ravault et al. 2005; Zeghnoun et al. 2005; Fuhrman et al. 2007; Motreff et al. 2013; Caillaud et al. 2015).

Comparing the studies to each other, one must keep in mind the use of different methodologies. Both the statistical methods and disease characterization vary widely between studies. In New York, allergy medications sold Over The Counter (OTC) were used (Sheffield et al. 2011). While this strategy permitted to catch mild cases who do not seek physician’s diagnosis, it also goes hand in hand with a loss of specificity by the inclusion of people with a wrong self-diagnosis or an influence of advertisements and promotions. In the Netherlands, all purchases of an oral antihistamine or a local anti-allergic drug were considered, as well as other ATC groups (Van Vliet and Tobi 2012). Moreover, data were aggregated to the weekly level in order to avoid the discrepancy between weekdays and weekends. Such an approach is valid, albeit costly in terms of precision. In French studies, cases were defined as the combined prescription of an oral antihistamine drug with a local anti-allergic drug (Ravault et al. 2005; Zeghnoun et al. 2005; Fuhrman et al. 2007; Motreff et al. 2013; Caillaud et al. 2015). For this study, allergic rhinitis cases were identified by the use of at least one medication belonging to the ATC category called “Antihistamines for systemic use”. This choice was, among others, supported by a study carried out in Belgium showing that oral antihistamines were the most frequently prescribed drugs (82.2% of patients received such a treatment) (Van Hoecke et al. 2006). Attempts were made to apply the French strategy on the Brussels case but this reduces the number of cases by a factor of four or more. Because the Poisson model is only valid if the daily counts are sufficiently large, this would have necessitated aggregating over age and gender groups, thus leading to less homogeneous age groups. Therefore, this definition would not have led to larger precision or power and information in subpopulation sensitivity would have been lost. This highlights the importance to consider the local context in terms of medical practices, refund policy and in generalizing/adapting the statistical methodology.

This is the first Belgian study investigating the short-term effect of pollen grains and fungal spores on medication sales. The Belgium capital constituted a very interesting field of study considering the high prevalence of allergic rhinitis in this country. Data used here distinguished themselves by their reliability and comprehensiveness. Indeed, they relied on a systematic recording organized by the national health insurance and concerned any Belgian residents. This system did not restrict to persons affiliated to a specific insurance programme or to a subset of pharmacies as for most of the previous works wherein population coverage ranged from 30% to 80% (Ravault et al. 2005; Sheffield et al. 2011). This present study also covered one of the longest period (7 years), increasing the statistical power of the analysis. Only one study investigated a longer time interval (10 years) (Caillaud et al. 2015).

Nevertheless, some limitations common to all studies analysing the relationship between aeroallergens and medication sales must be underlined. First, sales of reimbursed allergy drugs remain a proxy of allergic rhinitis morbidity due to pollen and fungal spores. Indeed, the specificity and the sensitivity of “Antihistamines for systemic use” medications to catch allergic rhinitis cases due to pollen and spores are not perfect. These drugs are for instance also included in many cough and cold preparations and can be used to treat urticarial skin rashes. Moreover, non-antihistamines such as intranasal steroids can also be prescribed for allergic rhinitis. More generally, the use of allergy medications is not limited to pollen and fungal spores: they can be prescribed for allergy to dust mite and pets’ dander. This issue is all the more significant in Europe where a large portion of the population is sensitized to these allergens (the median age–sex standardized prevalence reaches 21.7% for *Dermatophagoides pteronyssinus* and 8.8% for cat’s dander according to the ECRHS (Bousquet et al. 2007)). These allergens contribute to the non-zero background level observed in allergy medication sales. Besides, sales of reimbursed drugs lead to focus on people diagnosed and treated with reimbursed medications. This is likely to exclude from the analysis patients with mild symptoms who do not seek healthcare, use alternative therapies. Also, misdiagnosis and so inadequate treatment is possible, especially late winter when allergic rhinitis might be confounded with its infectious equivalent or cold. Furthermore, sales are not necessarily synonymous of symptoms. Indeed, they can correspond to purchase in prevention which might be a common practice among diagnosed people. Besides, one drug box may serve for several exposure periods. This might lead to underestimate real pollen impact. Second, an approximation is made regarding pollen and spore levels. Aeroallergen counts do not consider variations in distribution across the city, time spent outdoor by each person, etc. According to previous works, they tend to imperfectly estimate personal exposure but show a good correlation with patients’ symptomology (Riediker et al. 2000; Frenz 2001; O’Meara et al. 2004; Brito et al. 2011). In this study, all individuals lived within 10 km of the spore trap. Restriction of the analysis to a smaller area did not show sensitivity. Third, the statistical models currently available account for seasonal trends and multiple confounders. However, as the aeroallergens are introduced one at a time, they do not consider cross-allergenicity phenomena, overlap in pollination period. One could consider using multi pollen and spore models to produce more accurate relative risk estimates. However, large correlations between pollen concentrations exist (Spearman ρ > 0.7) and prevent the use of such analyses. Furthermore, no satisfactory statistical approach yet exists for the variety of synergies (interactions) that may result from the complex interplay between sequential exposures and administration of personal stock.

## Conclusion

This work relied on large national datasets which provided great information on allergic rhinitis management over a lengthy period. It highlighted the crucial role of Poaceae, *Betula*, *Carpinus*, *Fraxinus* and *Quercus* in allergic rhinitis morbidity in the BCR. Risks varied according to age and gender: the 19–64 years group was sensitive to the widest diversity of pollen and showed the highest relative risk. On the field, these conclusions could help to customize prevention programmes. With this in mind, it could be interesting to go into subpopulation sensitivity in depth considering for instance the impact of concomitant asthma or socioeconomic status. These kind of analyses could also be used in combination with clinical studies to monitor disease prevalence or severity in response to allergens and detect potential changes related to on-going environmental alterations (*Ambrosia*, *Artemisia* propagation, peak air pollution, etc.).

## Appendix

See Table 5.

**Table 5:** List of Drugs Considered for this Study

Substance	Brand name
Cetirizine	Cetirizine Sandoz
Cetirizine, -hydrochloride	Cetirizine Mylan
Cetirizine, -dihydrochloride	Cetirizine EG, Cetirizine UCB, Cetiriteva, Cetisandoz, Docceteri 10, Histimed, Hyperpoll, Zyrtec, Zyrtec Pi Pharma
Desloratadine	Aerieus
Dexchlorpheniramine, -maleate	Polaramine
Ebastine	Ebastine TEVA, Estivan, Estivan Lyo
Ketotifen	Ketitofen TEVA, Zaditen, Zaditen Retard
Levocetirizine, -dihydrochloride	Levocetirizine apotex, Levocetirizine EG, Levoceterizine Ratio, Levocetirizine Sandoz, Levocetirizine TEVA, Xyzall
Loratadine	Claritine, Loratadine EG, Loratadine Mylan, Loratadine Sandoz, Loratadine Teva, Rupton
Mizolastine	Mizollen
Promethazine hydrochloride	Phenergan
Rupatadine, fumaraat	Rupatall
